# The major leucyl aminopeptidase of *Trypanosoma cruzi *(LAPTc) assembles into a homohexamer and belongs to the M17 family of metallopeptidases

**DOI:** 10.1186/1471-2091-12-46

**Published:** 2011-08-23

**Authors:** Gloria Cadavid-Restrepo, Thiago S Gastardelo, Eric Faudry, Hugo de Almeida, Izabela MD Bastos, Raquel S Negreiros, Meire M Lima, Teresa C Assumpção, Keyla C Almeida, Michel Ragno, Christine Ebel, Bergmann M Ribeiro, Carlos R Felix, Jaime M Santana

**Affiliations:** 1 Department of Cell Biology, The University of Brasília, Brasília, 70910-900, Brazil; 2 Department of Bioscience, Universidad Nacional de Colombia, Núcleo El Volador, Calle 59A No 63-20, Medellín, Colombia; 3 Biology of Cancer and Infection IRTSV CEA, UMR1036 INSERM, Grenoble, France; 4 Bacterial Pathogenesis and Cellular Responses ERL5261, CNRS, Grenoble, France; 5 Université Joseph Fourier, Grenoble, France; 6 Faculty of Ceilândia, The University of Brasília, Brasília, 70910-900, Brazil; 7 Laboratory of Malaria and Vector Research, National Institutes of Health, Rockville, MD 20852, USA; 8 CEA, Institut de Biologie Structurale, F-38027 Grenoble, France; 9 CNRS, UMR 5075, Institut de Biologie Structurale, F-38027 Grenoble, France

## Abstract

**Background:**

Pathogens depend on peptidase activities to accomplish many physiological processes, including interaction with their hosts, highlighting parasitic peptidases as potential drug targets. In this study, a major leucyl aminopeptidolytic activity was identified in *Trypanosoma cruzi*, the aetiological agent of Chagas disease.

**Results:**

The enzyme was isolated from epimastigote forms of the parasite by a two-step chromatographic procedure and associated with a single 330-kDa homohexameric protein as determined by sedimentation velocity and light scattering experiments. Peptide mass fingerprinting identified the enzyme as the predicted *T. cruzi *aminopeptidase EAN97960. Molecular and enzymatic analysis indicated that this leucyl aminopeptidase of *T. cruzi *(LAPTc) belongs to the peptidase family M17 or leucyl aminopeptidase family. LAPTc has a strong dependence on neutral pH, is mesophilic and retains its oligomeric form up to 80°C. Conversely, its recombinant form is thermophilic and requires alkaline pH.

**Conclusions:**

LAPTc is a 330-kDa homohexameric metalloaminopeptidase expressed by all *T. cruzi *forms and mediates the major parasite leucyl aminopeptidolytic activity. Since biosynthetic pathways for essential amino acids, including leucine, are lacking in *T. cruzi*, LAPTc could have a function in nutritional supply.

## Background

The kinetoplastid protozoan *Trypanosoma cruzi *is the aetiological agent of Chagas disease, a debilitating chronic infection that is highly prevalent in Latin America and a worldwide concern because of human migration. Its complex life cycle includes four main distinctive developmental stages. In the insect vector, blood trypomastigotes transform into dividing epimastigotes that, after growth, undergo differentiation into the infective metacyclic trypomastigotes. In the cytoplasm of mammalian cells, metacyclic trypomastigotes transform into amastigotes that multiply and differentiate into trypomastigotes, which can reach the blood stream upon host cell disruption [1]. There is no vaccine for prevention of Chagas disease and the drugs currently employed in treatment strategies are toxic and ineffective in inhibiting disease progression to the chronic phase, resulting in thousands of deaths each year. In this context, the molecular and functional characterization of *T. cruzi *targets is necessary for the development of new chemotherapics for Chagas disease [2,3].

Peptidase activities are implicated in many aspects of the physiology of organisms, as well as in pathogen-host cell interface and pathogenesis, and are thus considered good drug targets [4,5]. *T. cruzi *growth, differentiation, dissemination through host tissues and infection of mammalian cells are highly dependent on proteolytic activities. The genome of *T. cruzi *contains many genes homologous to those encoding proteases which are considered virulence factors in other pathogens. However, only a few of these enzymes have been functionally characterized to date. Among them, cathepsin L, which is known as cruzipain, is associated with both *T. cruzi *development and infection [6,7]. Oligopeptidase B and POP Tc80, which are members of the prolyl oligopeptidase family of serine proteases, play important roles during parasite entry into mammalian cells [8-10]. *T. cruzi *differentiation depends on proteasome activity, while antibodies against surface metalloproteases partially block infection by trypomastigotes [11,12]. Additionally, the cysteine protease cathepsin B, a serine carboxipeptidase, and, more recently, two cytosolic metallocarboxypeptidases, a serine oligopeptidase and two aspartyl proteases have been biochemically characterized [13-17]. In contrast, the study of aminopeptidases has been limited to the detection of such activity in cell extracts of *T. cruzi *epimastigotes [18].

Leucyl aminopeptidases (EC 3.4.11.1; LAPs) are metalloaminopeptidases that catalyse the removal of N-terminal amino acid residues, preferentially leucine, from proteins and peptides. LAPs comprise a diverse set of enzymes with different biochemical and biophysical properties, are found in animals, plants and microorganisms, and play important roles in physiological processes, such as the catabolism of endogenous and exogenous proteins, peptide and protein turnover and processing, modulation of gene expression, antigen processing and defence [19]. LAPs in the peptidase family M17 show two unrelated domains, with the active site in the C-terminal domain. Their activities require two metal ions, are found to be maximal at neutral/basic pH, and are sensitive to bestatin and amastatin [20]. Because of their essential functions in the life cycle of microorganisms such as *Plasmodium*, *Fusobacterium nucleatum*, and the African trypanosome, LAPs are emerging as novel and promising pathogen targets for drug design [21-23]. Furthermore, LAPs are considered potential vaccine candidates, as evidenced by specific immune protection of sheep and cattle against fascioliasis [24].

The aim of this study was to examine leucyl aminopeptidase activity present in the developmental forms of *T. cruzi*. We report the identification, purification and characterization of the major leucyl aminopeptidolytic activity mediated by a hexameric 330-kDa leucyl aminopeptidase of *T. cruzi *(LAPTc), whose assembly does not depend on interchain disulfide bonds. Its molecular and enzymatic properties lead us to classify LAPTc as an archetypal member of the peptidase family M17. Different from its recombinant form that is alkaline and thermophilic, LAPTc purified from epimastigotes is neutral, mesophilic, and retains its oligomeric structure after losing activity at 80°C. Our data suggest that the enzyme localizes within vesicles in the cytoplasm of epimastigostes, trypomastigotes and amastigotes of *T. cruzi*. We postulate that LAPTc might be a potential target for the development of new drugs to treat *T. cruzi *infections.

## Results

### *T. cruzi *enzyme extract mediates hydrolysis of the aminopeptidase substrate Leu-AMC

**Figure 1:**
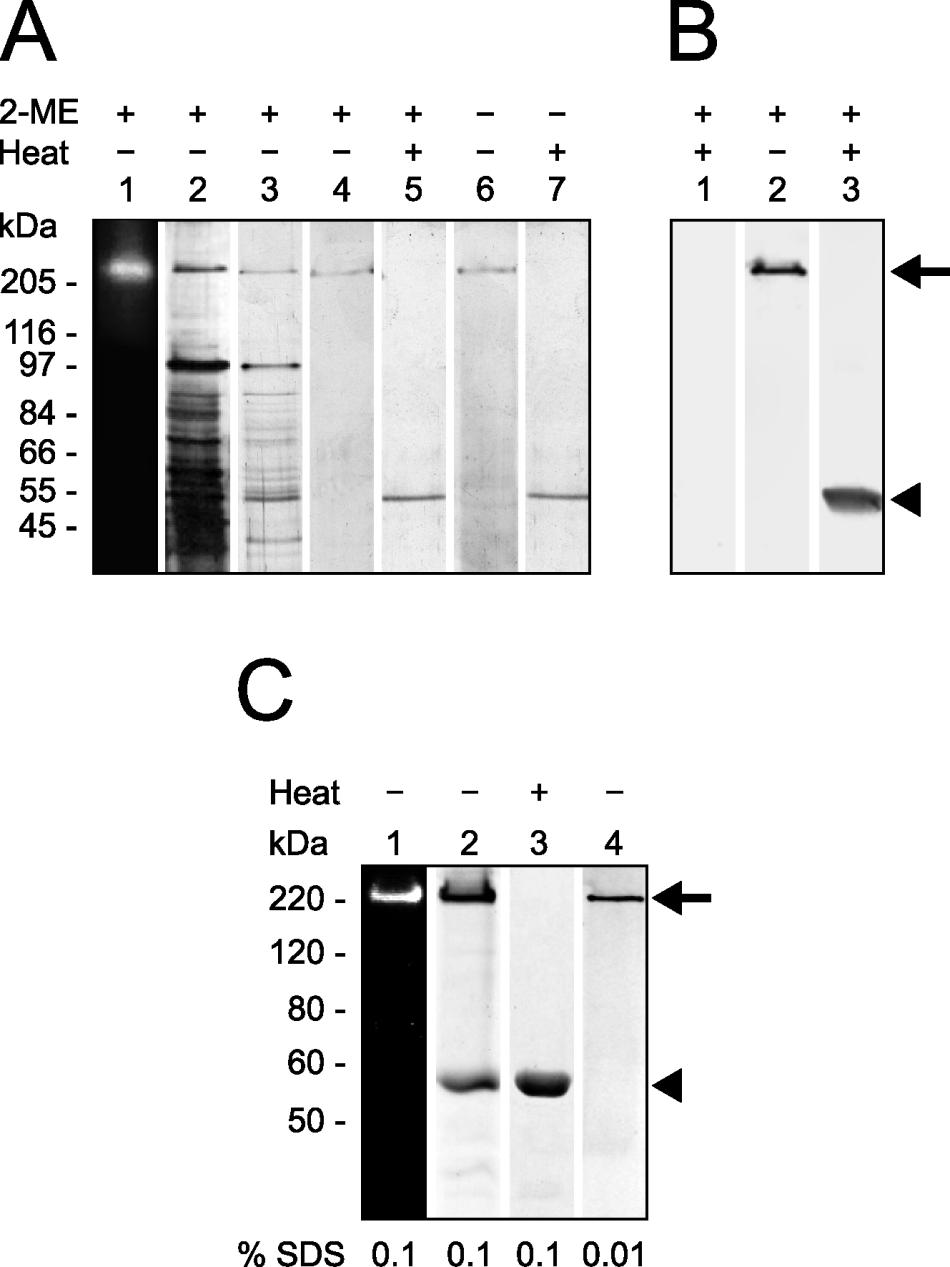
**Leucyl aminopeptidase behaves as an approximately 200-kDa homo-oligomer under electrophoresis and lacks interchain disulfide bonds**. **(A)**, gel zymographic experiments of total proteins of *T. cruzi *show a leucyl aminopeptidolytic activity upon Leu-AMC under UV light (*lane 1*). The same gel was Coomassie-stained (*lane 2*). Two purification steps of native LAPTc: DEAE-Sepharose column fraction (*lane 3*), Superose-6 column fraction without (*lane 4*) or with (*lane 5*) previous boiling, and under reducing (*lanes 4 *and *5*) or nonreducing (*lanes 6 *and *7*) conditions. **(B)**, western blot analysis of LAPTc after 8% SDS-PAGE of epimastigote total proteins with (*lanes 1 *and *3*) or without (*lane 2*) previous boiling, showing both oligomeric (*arrow*) and monomeric (*arrowhead*) forms of the enzyme. **(C)**, purified rLAPTc was subjected to 8% SDS-PAGE zymography without previous boiling of the sample (*lane 1*). The same gel was subsequently stained with Coomassie Blue (*lane 2*). The recombinant enzyme was also subjected to PAGE in the presence of 0.1 (*lane 3*) or 0.01% SDS (*lane 4*). Arrow and arrowhead indicate oligomeric and monomeric forms of the enzyme, respectively.

The sequencing of *T. cruzi *genome revealed genes coding for putative peptidases that mediate aminopeptidolytic activities http://www.tcruzidb.org/tcruzidb/home-ori.jsp. To identify such activities in *T. cruzi*, we prepared enzyme extract from epimastigoste forms of the parasite and incubated it with Leu-AMC, *N*-CBZ-Leu-AMC, Pro-AMC or Asp-AMC. Under these experimental conditions, only Leu-AMC was hydrolyzed by the enzyme extract from epimastigotes, with a calculated specific enzymatic activity of 45.86 ± 3.75 mU/mg of protein. The values of specific enzymatic activity obtained with enzyme extracts prepared from trypomastigotes and amastigotes were 30.56 ± 3.00 and 56.46 ± 4.62 mU/mg of protein, respectively. These results may suggest that this enzymatic activity is differentially regulated in the parasitic forms. Since the enzyme extract failed to hydrolyze *N*-CBZ-Leu-AMC (the blocked version of Leu-AMC), the hydrolysis of Leu-AMC may be mediated by a leucyl aminopeptidase. The molecular mass of the enzyme displaying such activity was estimated by gel enzymography. For this assay, the proteins present in the enzyme extract were separated by SDS-PAGE, followed by gel washing for enzymatic activity recovery and incubation in reaction buffer containing Leu-AMC. A single fluorescent band just above 200 kDa molecular mass was revealed which corresponded to free AMC released upon hydrolysis of the substrate (Figure 1A, *lane 1*). The enzymatic activity on Leu-AMC was observed to co-localize with a protein band upon staining of the same gel (Figure 1A, *lane 2*).

### Leucyl aminopeptidase is assembled into a homo-oligomer

The enzyme mediating hydrolysis of Leu-AMC was purified to homogeneity from freshly prepared enzyme extract by a combination of ion exchange and size exclusion chromatography with final yield and purification factor of 65 and 42%, respectively. The leucyl aminopeptidase activity was eluted from a DEAE-Sepharose column from 0.54 to 0.63 M NaCl as a single peak of activity. The active fractions were further purified on a Superose-6 HR column; again a single 300-kDa peak of enzymatic activity was observed (results not shown), which indicates that, under the conditions of this experiment, only one peptidase in the enzyme extract prepared from *T. cruzi *epimastigotes displays hydrolysis of Leu-AMC. The lack of hydrolysis of fluorogenic protease substrates such as Pro-AMC, Asp-AMC, *N*-CBZ-Leu-AMC, Gly-Phe-AMC, Gly-Arg-AMC, and Gly-Pro-AMC, as well as the protein substrates bovine serum albumin, immunoglobulin G and gelatin (not shown) suggests that the purified aminopeptidase displays narrow-spectrum activity.

The electrophoretic profiles of enzymatic active fractions on Leu-AMC obtained at each purification step are shown in Figure 1A (*lanes 3 *and *4*). A single Coomassie-stained band of approximately 200 kDa is seen after 8% SDS-PAGE under reducing conditions without previous boiling of the sample, which indicates that the aminopeptidase was obtained with high purity. However, when the purified enzyme was heated to 100°C for 5 min prior to electrophoretic analysis under reducing conditions, only a single 55-kDa protein band was revealed upon staining of the gel (*lane 5*). These data indicate that this active leucyl aminopeptidase is assembled into a homo-oligomer formed by monomers of about 55 kDa. We could not assess whether the monomer mediates enzymatic activity because it was only obtained upon boiling the oligomeric aminopeptidase.

To investigate the involvement of inter-monomer disulfide bonds in the stabilization of the aminopeptidase's oligomeric state, purified protein, previously boiled or not, was subjected to SDS-PAGE under reducing or nonreducing conditions (Figure 1A, *lanes 4 *and *5 versus lanes 6 *and *7*, respectively). The presence of a reducing agent did not change the electrophoretic migration pattern of the purified aminopeptidase (*lanes 1-5*). In contrast, high temperature induced monomerization of the protein oligomeric form; the active oligomer was only seen in the gels where the samples had not been previously heated to 100°C (*lanes 1-4 *and *6*), while its 55-kDa monomer was revealed upon sample boiling (*lanes 5 *and *7*). Since monomerization of the endogenous aminopeptidase occurs regardless of the presence of reducing conditions, we conclude that inter-monomer disulfide bonds do not take part in the assembly of the active oligomer.

### Mass spectrometry identification of the purified aminopeptidase

The molecular identity of the aminopeptidase with specificity for Leu-AMC was assessed by peptide mass fingerprinting. For this experiment, the purified native enzyme was digested with trypsin and the resulting peptides were subjected to MALDI-TOF analysis. Mass values of the detected peptides were compared to those theoretically deduced from sequences deposited in the database. Ten peptides showed mass matches to peptides obtained from theoretical digestion of the predicted leucyl aminopeptidase of *T. cruzi *EAN97960 (Table 1), which is encoded by gene ID Tc00.1047053508799.240 http://www.tcruzidb.org/tcruzidb/home-ori.jsp. This leucyl aminopeptidase gene (*laptc*) encodes for a 520-amino acid protein with a calculated molecular mass of 55,891 Da, and whose sequence does not comprise a predicted peptide signal. These observations correlate well with our experimental data showing that the purified enzyme displays leucyl aminopeptidase activity.

**Table 1:** Identification of *T. cruzi *protease by peptide mass fingerprinting

Tryptic peptide masses (Da)		Identified amino acid sequences
	
**Experimental data**^**a **^ (in-gel digestion)	*T. cruzi *aminopeptidase (*in silico *digestion)	
2,193.39	2,192.38	^36^K.THTAGLASTFVVILGTHAQLR.E^56^
2,749.63	2,748.62	^36^K.THTAGLASTFVVILGTHAQLR↓EDALK.E^61^
1,764.03	1,763.02	^62^K.ELPFYCPAVAEAIQR.V^76^
3,011.58	3,010.57	^182^R.LTVVFTPAPNPSPSELVVVATSTQLCQR.L^209^
2,437.36	2,436.35	^210^R.LVDAPTNLLNTATFAEVAQSYAK.E^232^
1,742.99	1,741.98	^310^R.DMGGAAAVFCGFLTAVR.L^326^
1,977.11	1,976.10	^412^R.HAGIFVNDEEEELSFLK.A^428^
2,590.34	2,589.34	^432^R.VSGETCFPVLYCPEYHVTEFR.S^452^
1,538.92	1,537.92	^504^K.ATGFGPALLMEYLR.N^517^
1,554.90	1,553.89	^504^K.ATGFGPALLMEYLR.N^517^

^a ^Masses are MH^+ ^monoisotopic, with accuracy up to 0.2 Da. The enzyme was digested with trypsin, and masses of resulting peptides were determined by MALDI-TOF mass spectrometry and compared to theoretical ones produced by *in silico *digestion of proteins found in the database (NCBInr). An oxidation of methionine is indicated as M. A missed cleavage is indicated as ↓.

**Figure 2:**
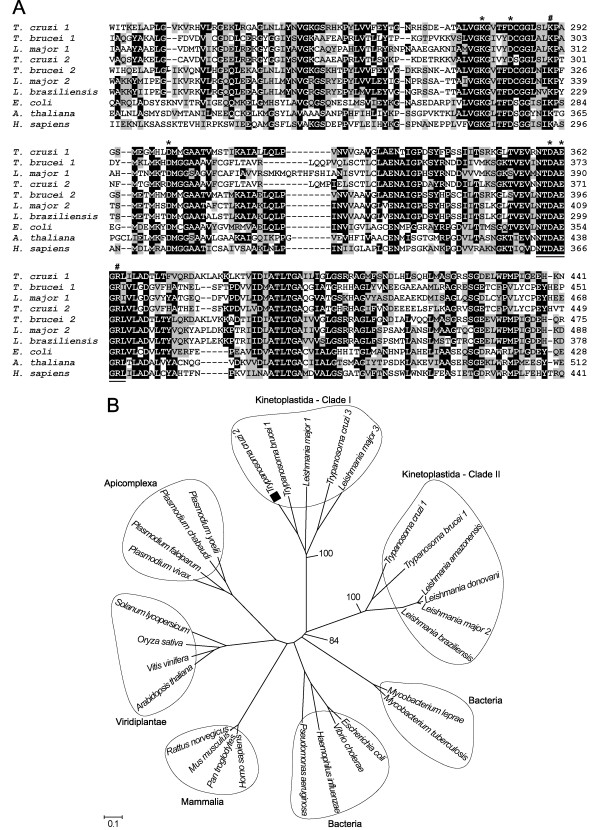
**Sequence comparison and phylogenetic relationship of LAPTc to other members of the M17 family of metallopeptidases**. **(A)**, Multiple C-portion amino acid sequence alignments of different LAPs. Amino acid sequences from the conserved C-terminal region of LAPs were aligned by the ClustalX program. Amino acids marked in black show 50% identity and those in gray show 50% similarity. Putative metal binding sites (*), catalytic site (#), and M17 signature (underlined) are indicated. Sequences were obtained from the protein database of the National Center for Biotechnology Information (NCBI) under the following accession numbers: EAN87580.1 (*T. cruzi *1) and EAN97960.1 (*T. cruzi *2), EAN79621.1 (*T. brucei *1) and AAX70152.1(*T. brucei *2), CAJ02694.1 (*L. major *1) and AAL16097.1(*L. major *2), CAM36610.1 (*L. braziliensis*), YP_672349.1 (*E. coli*), NP_194821.1 (*A. thaliana*), AAD17527.1 (*H. sapiens*). **(B)**, Phylogenetic relationship of LAPTc to other LAPs of different organisms. 29 full-length sequences, derived from the nonreduntant (NR) protein database of the NCBI (listed in Experimental Procedures), were aligned by the ClustalX program, and the phylogram was generated with the Mega package after 10,000 bootstraps with the neighbor joining (NJ) algorithm. The bar scale at the bottom represents 10% amino acid substitution per site. LAPTc is indicated by a closed square.

According to sequence homology, this leucyl aminopeptidase of *T. cruzi *(LAPTc) belongs to the metallopeptidase M17 family, also known as the leucyl aminopeptidase family [25]. It shares 34 to 66% identity to other members of the M17 family, including assigned and unassigned leucyl aminopeptidases of kinetoplastidae parasites. Multiple amino acid sequence alignments (Figure 2A) also revealed that the C-terminal portion is the most conserved region in this family, reaching 72% identity and 83% similarity between *T. cruzi *and *T. brucei*. The sequence of LAPTc comprises the highly conserved active site (Lys^299 ^and Arg^373^), metal binding residues (Lys^287^, Asp^292^, Asp^310^, Asp^369 ^and Glu^371^) and the signature NTDAEGRL sequence of the M17 family [26]. The phylogenetic tree shows divergent groups of proteins that cluster by phylum or class (Figure 2B). It is interesting to note that bacteria, mammalia, viridiplantae and apicomplexa have an indication of a common ancestor with a strong bootstrap support. Kinetoplastid sequences are divided in two defined clades, again with very strong bootstrap support. One group of kinetoplastids comprises sequences annotated as aminopeptidases and the other group contains sequences assigned as leucyl aminopeptidases. Although these two clades are members of the M17 family, their sequence divergence indicates that the ancestral trypanosomatid giving origin to both *Leishmania *and *Trypanosoma *already contained these two enzymes.

### LAPTc assembles into a hexamer

The recombinant active and soluble form of LAPTc was produced in *E. coli *containing a His-tag at its N-terminus. It was purified by affinity chromatography on a nickel column upon elution with 200 mM imidazol and then submitted to size exclusion chromatography. The activity co-migrates with the main protein peak of 320 kDa (not shown) that was submitted to SDS-PAGE analysis. In-gel enzymography of the gel showed that only a 220-kDa protein band mediates enzymatic activity on Leu-AMC when PAGE was carried out without previous heating of the sample and in the presence of 0.1% SDS (Figure 1C, *lane 1*). Protein bands of about 220 and 55 kDa were revealed upon staining of the same gel (Figure 1C, *lane 2*). Under the same experimental conditions, sample boiling resulted in complete monomerization of rLAPTc (Figure 1C, *lane 3*). Unlike its endogenous form that conserves an oligomeric structure in the presence of 0.1% SDS (Figure 1A), rLAPTc is very sensitive to this detergent and is only entirely seen as an oligomer in the presence of SDS as low as 0.01% (Figure 1C, *lane 4*). These data show that, regardless of their sensitivity to SDS, both endogenous and recombinant forms of LAPTc behave the same when submitted to PAGE and size exclusion chromatography.

**Figure 3:**
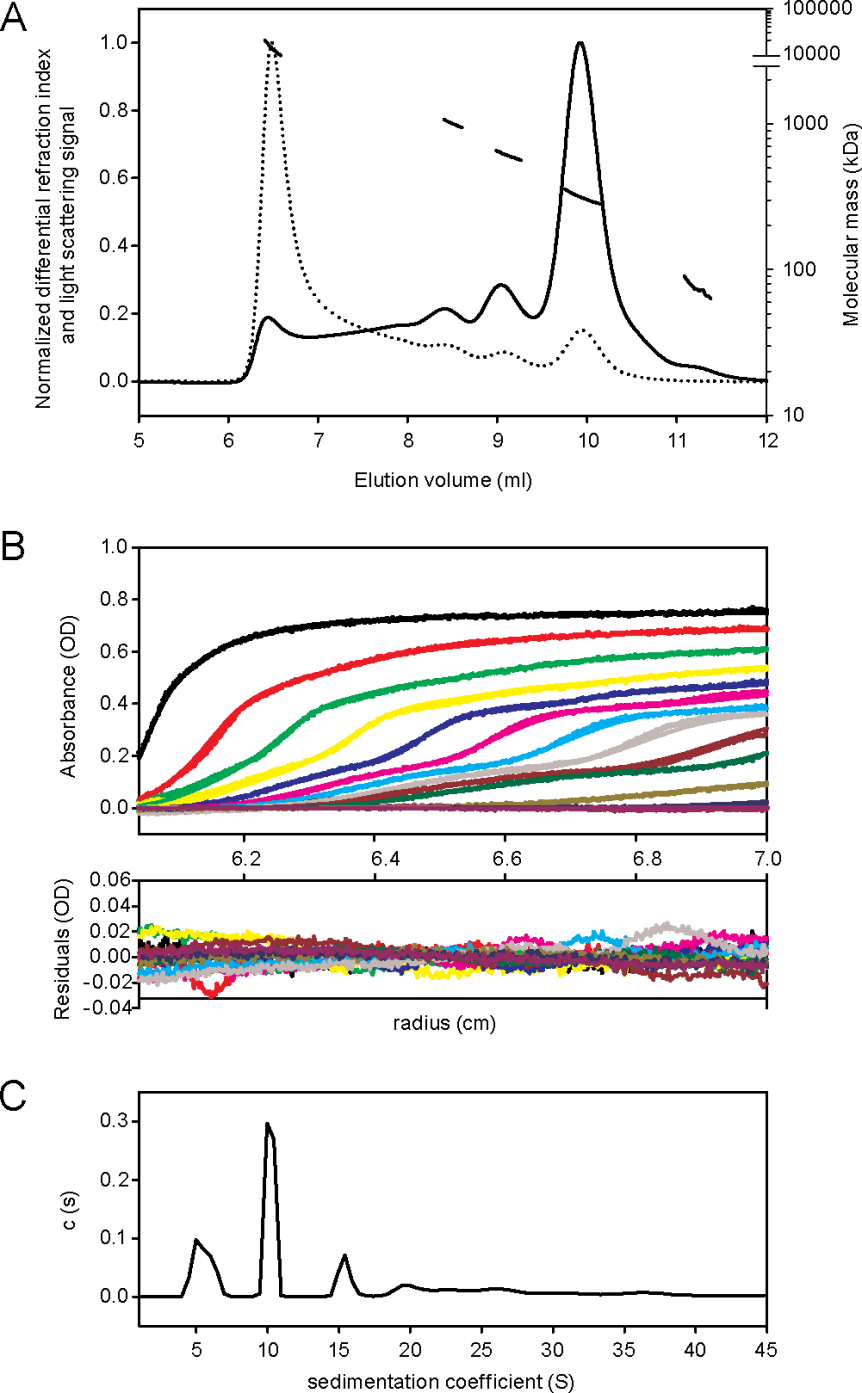
**LAPTc assembles into a hexamer**. **(A)**, SEC-MALLS data obtained with 20 μl rLAPTc at 10 mg/ml (170 μM). Superposition of concentration signal (differential refractive index, continuous line), light scattering signal (dotted line) and molecular mass calculation (discontinuous line). For clarity, the differential refractive index and light scattering signals are normalized. **(B)**, superposition of experimental (dots) and fitted (continuous line) AUC profiles corrected for all systematic noise for LAPTc at 56 μM (upper panel). Superposition of the differences between the experimental and fitted curves (lower panel). The fit was obtained from the c(s) analysis of the program SEDFIT. **(C)**, corresponding c(s) distribution in the range 1-45 S.

To solve the divergence in its molecular mass determination, we further submitted affinity chromatography purified rLAPTc to SEC-MALLS and to analytical ultracentrifugation analysis. MALLS measurements allow the molecular mass of macromolecules in solution to be calculated, taking into account the absolute concentrations obtained with a differential refraction index detector. The elution profile showed the presence of five resolved peaks corresponding to different oligomeric species eluting at 6.5, 8.5, 9, 10 and 11.2 ml (Figure 3A, *continuous line*). The main protein peak was eluted at 10 ml and represents 45% of the mass recovery. As expected, light scattering measurements (Figure 3A, *dotted line*) exhibited higher signal for the larger species eluting first, given that light scattering is directly related to the concentration and molecular mass of the observed objects. Molecular mass calculations (Figure 3A, *discontinuous line*) revealed that the first protein peak (6.5 ml) corresponds to highly aggregated species with molecular masses above 10,000 kDa. The peaks eluting at 8.5, 9, 10 and 11.2 ml correspond to oligomers of 1025, 625, 314 and 176 kDa, respectively. In conclusion, SEC-MALLS experiments showed that rLAPTc is predominantly assembled into a 314-kDa oligomer, but other minor species, not detectable during the purification procedure of the endogenous enzyme, also co-exist (Table 2).

**Table 2:** Summary of MALLS and AUC experiments

SEC-MALLS			Calibrated SEC-AUC			N° of subunits
		
Elution volume (ml)	Recovery mass fraction (%)	Molecular mass (kDa)	Stoke radius (nm)	Sedimentation coefficient (S)	Molecular mass (kDa)	
8.5	11	1025	ND	19.5	ND	17.5^a^
9	13	625	6.8	15.3	593	10.1^b^
10	45	314	5.7	10.2	330	5.6^b^
11.2	4	176	ND	5.1	ND	3^a^

^a ^From SEC-MALLS; ^b ^From SEC-AUC

The hexameric form of LAPTc was confirmed by analytical ultracentrifugation (AUC), a versatile and powerful tool for the identification of oligomeric states and the determination of protein molecular masses [27]. Figure 3B shows the experimental and fitted sedimentation velocity profiles obtained at 56 μM by monitoring the absorbance at 295 nm. The derived sedimentation coefficient distribution (Figure 3C) exhibits four main species sedimenting at 5.1, 10.2, 15.3 and 19.5 S (*s_20,w _*= 7.0, 13.9, 20.9 and 26.6 S, respectively). The *s*-value depends on the molar mass, *M*, and Stokes radius, *R*_S_, of the particle, according to the Svedberg equation: $s=M\left(1-\rho \bar{v}\right)∕\left(N_{\text{A}}6\pi \eta R_{S}\right)$. To calculate the corresponding molecular masses, calibrated size exclusion chromatography (SEC) was performed with the same samples, giving Stokes radii for the two main species eluting at 9 and 10 ml of 6.8 and 5.7 nm, respectively. The combination of the *s*-values of 15.3 and 10.2 S with *R*_S _= 6.8 and 5.7 nm (SEC-AUC method) gives the estimates for the species of *M *= 593 and 330 kDa, respectively (Table 2), confirming the results obtained by SEC-MALLS. Considering the monomer molecular mass deduced from the sequence, 58.7 kDa, the calculated number of subunits present in the main species eluting at 10 ml is 5.6, suggesting a pentamer or, more likely, a hexamer. Taking into account 5 or 6 as the number of subunits, the inferred *R*_S _values from the Svedberg equation are 5.1 and 6.1 nm, which correspond to frictional ratios of 1.16 and 1.31, respectively. These are within the values expected for globular proteins. However, the frictional ratio obtained for the pentamer hypothesis is somewhat low for a 330-kDa protein. Thus, these data indicate that the main rLAPTc species is a hexamer. The sedimentation distributions of rLAPTc at 170, 56 and 10 μM present the same main features. However, the ratio of hexamer to trimer decreases when the concentration of the enzyme goes from 56 to 10 μM. In addition, at concentrations as high as 170 μM the amount of large aggregates increases significantly. Our data thus show a complex equilibrium among different multimers depending on enzyme concentration.

### Recombinant and native forms of LAPTc display distinct activity features

**Figure 4:**
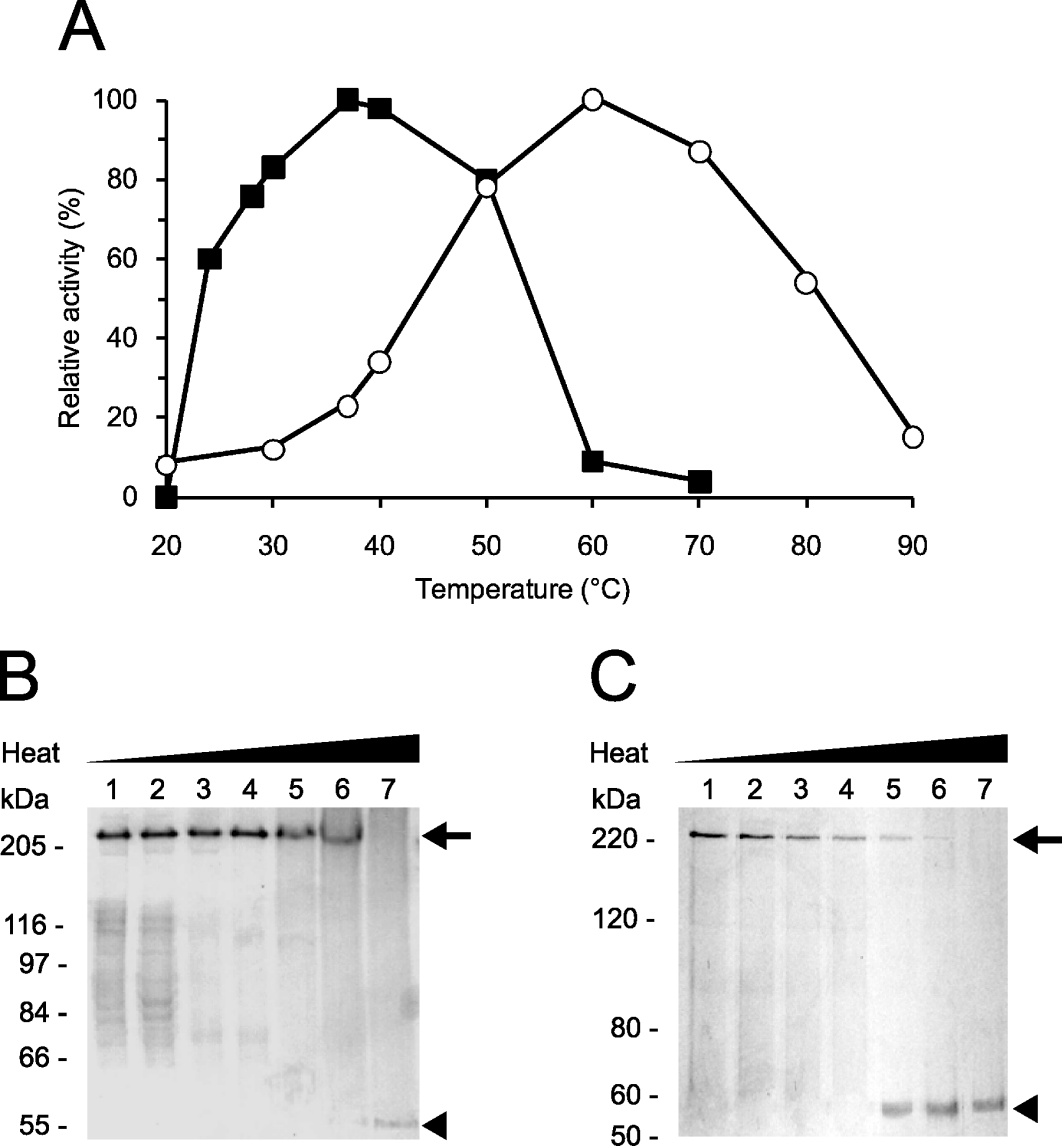
**Optimal temperature for activity versus eletrophoretic migration pattern of LAPTc**. **(A)**, both native (*solid squares*) and recombinant (*open circles*) purified enzymes were incubated with Leu-AMC in reaction buffer at different temperatures for 15 min and the AMC released was measured as described in Experimental Procedures. Standard deviations were less than 10%. LAPTc **(B) **or rLAPTc **(C) **was incubated at 20 (*lanes 1*), 37 (*lanes 2*), 50 (*lanes 3*), 60 (*lanes 4*), 70 (*lanes 5*) 80 (*lanes 6*) or 100°C (*lanes 7*) for 10 min in reaction buffer, followed by 8% SDS-PAGE analysis. Oligomeric (*arrow*) and monomeric (*arrowhead*) forms of the enzymes are indicated. Gels were Coomassie-stained.

The influence of pH on the activity of purified LAPTc and rLAPTc was determined. Maximal specific activity for the native enzyme was measured at pH 7.0 (data not shown). At pHs 6.0 and 8.0 the recorded specific activities were 45% of that measured at pH 7.0, whereas at pHs 5.0 and 9.0 the enzyme was shown to be inactive. Conversely, for rLAPTc the optimal pH is 8.0; at pH 7.5 and 9.0 the enzyme loses 60 and 75%, respectively, of its activity recorded at pH 8.0. These data demonstrate that LAPTc has a strong dependence on neutral pH, whereas its recombinant form displays maximal activity at pH 8.0. The optimum temperature for LAPTc activity on Leu-AMC was shown to be 37°C. Nevertheless, the enzyme retained 85% of its activity over a broad temperature range 30-50°C), suggesting stability and absence of regulation depending on the *T. cruzi *host (Figure 4A). In contrast, rLAPTc exhibits a distinct activity profile at different temperatures; specific activity measured at 37°C corresponded to only 25% of the recorded maximal activity observed at 60°C (Figure 4A). These data indicate that the native enzyme is mesophilic, whereas its recombinant form produced in *E. coli *is thermophilic. To study the thermostability of LAPTc, hydrolysis of Leu-AMC by native and recombinant forms of the enzyme was assayed at 37 or 60°C, respectively, after preincubation at different temperatures for either 15 or 240 min (Table 3). Under these experimental conditions, the enzymatic activity of LAPTc was not significantly modified after preincubation at 37°C for 240 min. However, preincubation at higher temperatures resulted in significant loss of enzymatic activity. rLAPTc was shown to be more stable than its native form, which correlates well with its higher optimal temperature of activity.

**Table 3:** LAPTc and rLAPTc thermostability

Preincubation temperature (°C)	**Enzymatic activity (% of control)**^**a**^			
	
	Preincubation for 15 min		Preincubation for 240 min	
		
	LAPTc	rLAPTc	LAPTc	rLAPTc
28	92	97	74	94
37	86	98	70	92
40	80	99	51	87
50	52	92	6	43
60	35	87	0	5
70	0	6	0	3
80	0	5	0	3

Purified LAPTc and rLAPTc were preincubated for 15 or 240 min at different temperatures, and then their activities on Leu-AMC were measured at 37 or 60°C, respectively. ^a ^Control consisted of enzyme activity test without prior incubation. Standard deviation was less than 11%.

The Michaelis-Menten constant (*K_m_*) and maximal velocity (*V*_max_) of LAPTc were determined according to the hyperbolic regression method. The endogenous enzyme has a *K_m _*value of 12.0 ± 0.8 μM Leu-AMC and its calculated catalytic constant (*k*_cat_) and catalytic efficiency (*k*_cat_/*K_m_*) are 12.47 ± 1.2 S^-1 ^and 1.04 ± 0.09 μM^-1^.S^-1^, respectively. *K_m_*, *k*_cat _and *k*_cat_/*K_m _*values for rLAPTc are 185.9 ± 17.0 μM, 34.84 ± 2.9 S^-1 ^and 0.19 ± 0.01 μM^-1^.S^-1^, in that order. These results show that native and recombinant LAPTc exhibit different kinetic parameters.

### LAPTc retains its oligomeric structure after losing activity

We asked whether the temperature-dependent enzymatic inactivation of LAPTc was due to monomerization of the oligomer. This question was addressed by incubating LAPTc for 15 min at different temperatures, followed by SDS-PAGE analysis. Although its enzymatic activity was almost completely lost at 60°C, the peptidase fully retained its oligomeric form upon preincubation up to 80°C (Figure 4B). Complete disassembly of the oligomer was achieved after boiling the sample, since LAPTc migrated as a single 55-kDa band in the gel. These data indicate that LAPTc keeps its oligomeric form after temperature-induced inactivation. On the other hand, rLAPTc monomerization as a function of temperature correlates well with its loss of activity (Figure 4C).

### LAPTc is a metalloaminopeptidase

The enzymatic activity of LAPTc on Leu-AMC was completely inhibited by 100 μM bestatin, while 250 μM 1,10-phenanthroline and 10 mM EDTA inactivated 83 and 45% of the peptidase activity, respectively (Table 4). LAPTc hydrolytic activity was not sensitive to PMSF, TLCK, E-64, leupeptin or pepstatin A. The activity of the enzyme previously inactivated by EDTA or 1,10-phenanthroline was potentiated by 0.4 mM Mn^2+ ^or Ca^2+ ^and restored to 80% of the control by Zn^2+ ^but not by Fe^2+ ^or Mg^2+^. In contrast, assay in the presence of Al^3+ ^or Co^2+ ^resulted in considerable inactivation of the enzyme (Table 4). Since LAPTc was specifically inhibited by metal chelating agents such as 1,10-phenanthroline, we consider it a member of the metalloprotease family.

**Table 4:** Inhibition pattern and cation dependence of LAPTc

Inhibitor	**Enzymatic activity (% of control)**^**a**^							
	
	No cation	**Mn**^**2+**^	**Ca**^**2+**^	**Zn**^**2+**^	**Fe**^**2+**^	**Mg**^**2+**^	**Al**^**3+**^	**Co**^**2+**^
10 mM EDTA	55	149	117	80	50	44	11	5
250 μM 1,10-phenanthroline	17	135	115	78	18	20	5	4
100 μM bestatin	3	ND^b^	ND	ND	ND	ND	ND	ND
2 mM PMSF	100	ND	ND	ND	ND	ND	ND	ND
10 μM E-64	98	ND	ND	ND	ND	ND	ND	ND
10 μM TLCK	97	ND	ND	ND	ND	ND	ND	ND
1 mM leupeptin	99	ND	ND	ND	ND	ND	ND	ND
200 μM pepstatin A	102	ND	ND	ND	ND	ND	ND	ND

^a ^LAPTc was incubated with inhibitor before enzymatic assay at 37°C with Leu-AMC. Peptidase incubated with EDTA or 1,10-phenanthroline was dialyzed against reaction buffer prior to incubation with 20 μM Leu-AMC and 0.4 mM MnCl_2_, CaCl_2_, ZnCl_2_, FeCl_2_, MgCl_2_, AlCl_3_, or CoCl_2_. Results represent means from three independent experiments carried out in triplicate. Standard deviations were less than 10%. ^b ^ND, not determined.

### LAPTc is expressed as an oligomer

**Figure 5:**
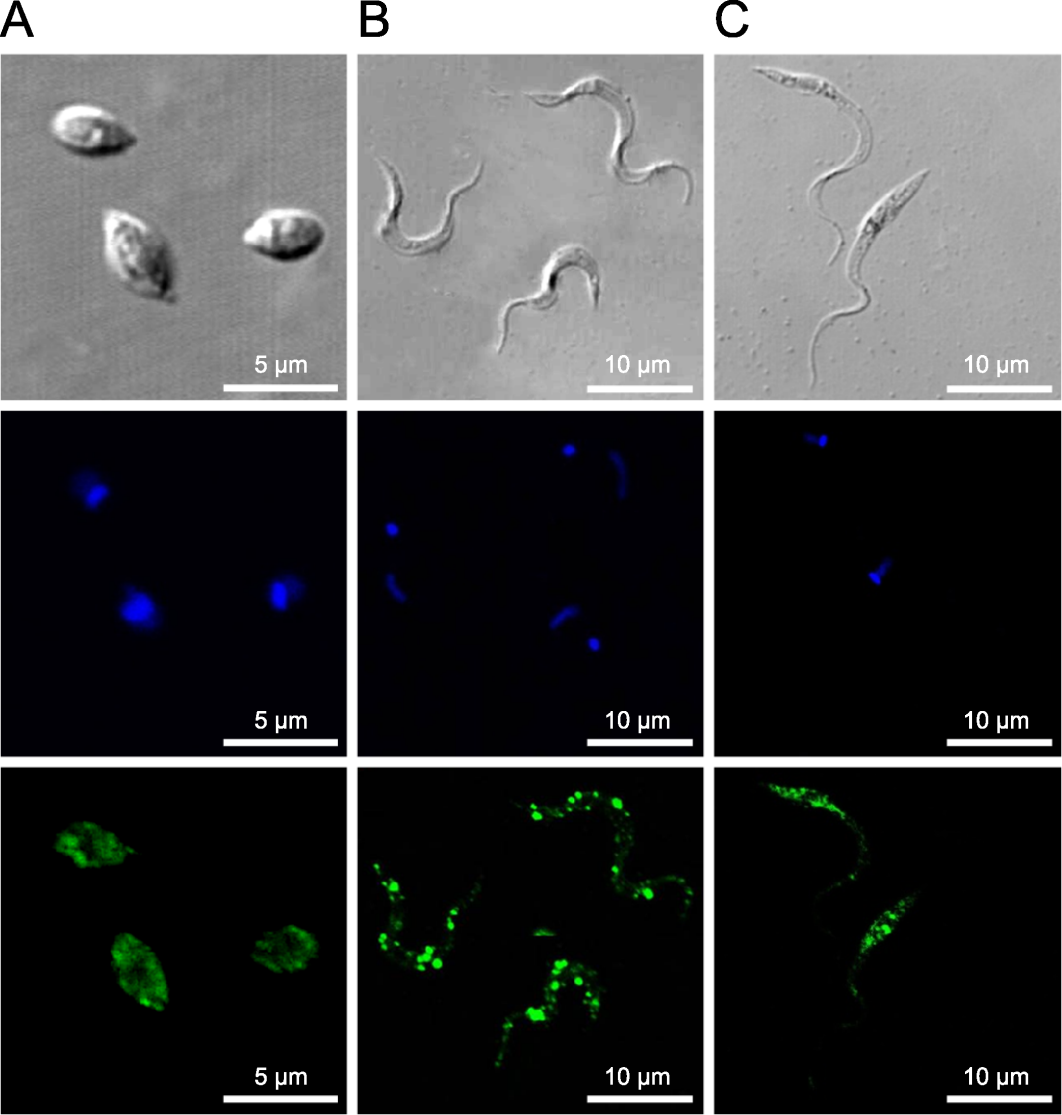
**Immunocytolocalization of LAPTc in the developmental forms of *T. cruzi***. Immunofluorescence analysis in amastigote **(A)**, trypomastigote **(B) **and epimastigote **(C) **forms. *Top row*, phase contrast microscopy; *middle row*, DAPI-stained parasite DNA (*blue*); *bottom row*, anti-LAPTc serum and developing with conjugate containing Alexa 488 (*green*).

To assay the expression of LAPTc by *T. cruzi*, total proteins of epimastigote cells were resolved in SDS-PAGE with or without previous heating to 100°C, transferred to a nitrocellulose membrane and probed with specific polyclonal antibodies raised against the purified enzyme (anti-LAPTc). Under the conditions of this experiment, anti-LAPTc reacted only with the oligomeric form of the enzyme where the proteins had not been boiled, and recognized its monomer upon heating epimastigote proteins. These results show that LAPTc is expressed as an oligomer by *T. cruzi *(Figure 1B). Anti-LAPTc antibodies were employed to determine where the enzyme localizes in the parasite through an immunofluorescence assay (Figure 5). Pre-immune serum was used in control experiments. The spot-like labeling pattern observed inside parasite cells suggest that LAPTc is located within vesicles in the cytoplasm of epimastigotes, amastigotes and trypomastigotes of *T. cruzi*. However, accurate localization of the enzyme in *T. cruzi *forms requires additional experiments.

## Discussion

*T. cruzi *whole genome sequencing has revealed 28 genes encoding putative aminopeptidases, amongst which there are three methionine, two aspartic, two puramycin-sensitive and three leucyl aminopeptidases of the M17 family. In the present work, we report the identification, purification and biochemical characterization of a major leucyl aminopeptidase activity of *T. cruzi*. The enzyme displaying this activity is the product of the Tc00.1047053508799.240 gene and was named LAPTc to designate its activity. Under the conditions examined, a single activity on Leu-AMC was observed either during the purification procedure or upon enzymography assay. These results suggest that LAPTc mediates a major leucyl aminopeptidase activity in *T. cruzi *epimastigotes. However, the absence of other such activities could be due to insolubility, low expression levels or instability of the products. For example, in contrast to other *T. cruzi *proteases such as oligopeptidase B and cathepsin B, the activity of POPTc80 cannot be detected by enzymographic assay due to irreversible denaturation [13,28,29]. The absence of detectable hydrolysis of BSA, gelatin, Pro-AMC and Asp-AMC substrates suggests that the activity of LAPTc is restrictive, which is in agreement with the specificities of M17 family members that are associated with degradation and processing of peptides and proteins by removing specific N-terminal amino acidic residues [30]. The differentiated expression of LAPTc activity by *T. cruzi *forms might be due to their different requirements of metabolites and processing of peptides and proteins. Epimastigotes live in axenic cultures, trypomastigotes are infective and found mainly in the blood and amastigotes divide inside mammalian host cells.

Aminopeptidases are widely distributed in animals, plants and microorganisms, and found in the extracellular millieu, in the cytoplasm, in many subcellular organelles, and as components of membranes [19]. These enzymes are either monomeric or multimeric, comprising one, two, four or six subunits. Although members of the M17 family have been mainly described as multimeric, some of them behave as monomeric. For example, recombinant LAPs of *Leishmania *spp. and *P. falciparum *exhibit a homohexameric structure, while those of *Haemaphysalis longicornis*, *Schistosoma monsoni *and *Schistosoma japonicum *seem to be monomeric enzymes [21,31-33]. In contrast, LAPTc displays an electrophoretic migration pattern corresponding to a homotetramer. However, it must be taken into account that some proteins display abnormal migration both in SDS-PAGE and size exclusion chromatography [34], and assembly of recombinant proteins might differ from that of their native forms. In addition, LAPTc three-dimensional structure may contribute to its fast migration since it was not heated before PAGE. Oligopeptidase B of *T. cruzi *also displays abnormal electrophoretic migration under the same experimental conditions [35]. Nevertheless, other enzymes such as *T. cruzi *cathepsin B and the hexameric leucyl aminopeptidase of *Borrelia burgdorferi *(TAP_Bb_) show the expected migration [13,36]. The hexameric nature of LAPTc was thus confirmed by analytical ultracentrifugation and MALLS assays, which are accurate techniques to determine molecular masses of macromolecules in the absence of any interaction with matrices or surfaces. As it has been observed for members of the M17 and M29 families, such as leucyl aminopeptidase of bovine lens, aminopeptidase A of *E. coli*, and TAP_Bb _[36-38], the oligomeric assembly of LAPTc does not require the presence of interchain disulfide bonds because monomerization occurs in the absence of a reducing agent. The oligomeric structures of these enzymes may be maintained through hydrogen bridges, Van der Waals and hydrophobic interactions as is observed for bovine lens aminopeptidase [26]. The advantage of multimeric over monomeric structures is still unclear, but it is possible that a quaternary structure allows not only hydrophobic regions to be hidden within the protein assembly but also the reduction of the macromolecule surface in contact with the medium, thus restraining the amount of water required to stabilize these proteins [39]. The association between enzymatic activity and multimeric structure of leucyl aminopeptidases suggests that either the active sites are formed at the subunit junctions or the three dimensional assembly stabilizes the active site of each monomer. The latter hypothesis is supported by the fact that the activity of bovine lens leucyl aminopeptidase depends on the stabilization of each monomer active site by the structure of the oligomer [26].

LAPTc comprises several distinctive characteristics of M17 leucyl aminopeptidases. In addition to conserved amino acid sequences, especially at the C-terminus, which contains two M17 Pfam domains, it also lacks the HEXXH signature found in the M1 family. Amino acid sequences deduced from cDNAs from many genomes have revealed amino acid sequence homologies in organisms as diverse as bacteria and mammals, particularly around residues involved in catalysis and metal ion binding [19]. As expected, LAPTc shows the highest identity with the M17 leucyl aminopeptidases of the kinetoplastids *L. major *and *T. brucei*, and less extensively with the unassigned aminopeptidase II of *T. cruzi*. Despite conservation of amino acid sequences, M17 members show variable pH and temperature optima. Although LAPTc is active over a broad range of temperatures, its activity shows a marked dependence on neutral pH, since at pH 6 and 8 the enzymatic activity is only 45% of that measured at pH 7. Furthermore, the enzyme is completely inactive at pH 5 and 9. It should be taken into account that an enzyme may mediate its activity over a broad pH range, depending on the substrate. Recombinant forms of *Leishmania *spp. LAPs show optimal activity at pH 8.0-8.5 on Leu-AMC and have zinc as a cofactor but its 62-kDa monomer does not mediate enzyme activity [31].

The distinguishable features between the two forms of the enzyme might be explained by folding differences, given that rLAPTc was produced in *E. coli *and LAPTc isolated from *T. cruzi*. The higher sensitivity of rLAPTc to SDS is in agreement with this hypothesis. This correlates well with observations that recombinant members of M17 assemble into active oligomers at 60-70°C and alkaline pHs [19]. Temperatures above 70°C, however, promote inactivation of the thermophilic TAP_Bb_, a member of the M29 family of metallopeptidases, through a transition from the hexameric to the monomeric state [36]. Since the active form of both endogenous enzymes lack interchain disulfide bonds, the oligomeric state of LAPTc is even more resistant to high temperatures than that of TAP_Bb_. However, the three-dimensional structure of LAPTc seems to unfold at 60°C, the optimal activity temperature of TAP_Bb_. In spite of displaying leucyl aminopeptidase activity, sequence identity among members of M29 and M17 families is almost absent. Resolution of three-dimensional structures of M29 peptidases may lead to a better understanding of the evolution and activity mechanism of the leucyl aminopeptidase superfamily members.

Members of M17 aminopeptidases have a broad range of functional properties beyond the degradation of peptides. In animals, plants and bacteria, these enzymes have been implicated in many physiological processes such as protein turnover, regulation of cell redox status, cataract development, MHC I-dependent antigen processing and presentation to cytotoxic T cells, nutritional supply, transcriptional regulation, protein and peptide maturation and defense [19,40]. A *P. falciparum *M17 peptidase is involved in amino acid uptake and regulation and, thus, is considered a virulence factor [21]. Arphamenine-A, an aminopeptidase inhibitor, restrains *in vitro *growth of *T. brucei*, a close relative of *T. cruzi *[23]. In this study, we show that LAPTc mediates the major leucyl aminopeptidase activity in *T. cruzi *extracts and, thus, it likely has important functions in physiological processes involving protein and peptide processing, degradation of proteins and amino acid recycling. *T. cruzi*, *Leishmania *spp. and *T. brucei *lack the biosynthetic pathways to synthesize the essential amino acids of humans, including leucine [41]. In spite of the metabolic relevance of amino acids for these parasites, their transport and recycling are poorly known. Although many putative amino acid transporter genes have been identified *in silico*, only arginine and proline transporters have been biochemically characterized in *T. cruzi *[42-44]. Considering that a biosynthetic pathway is missing, *T. cruzi *must acquire leucine through specific transport and/or recycling. Since amastigotes live and divide within host cells where the concentration of free amino acids is low, leucine aminopeptidases would play a major role in leucine supply to the parasite through hydrolysis of exogenous and endogenous proteins and peptides. Inactivation of LAPTc activity by specific inhibitors or through gene disruption may help reveal its functional properties and thus its importance to the host-*T. cruzi *interface.

## Conclusions

LAPTc is a 330-kDa homohexameric enzyme that mediates the major leucyl aminopeptidase activity in *T. cruzi*. Inter-monomer disulfide bonds do not take part in the assembly of the active oligomer. LAPTc is a member of the metallopeptidase M17 family or leucyl aminopeptidase family. It retains its oligomeric structure after losing activity and is expressed by all *T. cruzi *forms.

## Methods

### Parasites and preparation of enzyme extract

*T. cruzi *epimastigote, amastigote and trypomastigote forms from Berenice stock were cultured and purified as described previously [10]. Cell-free extracts were prepared from 100 ml of epimastigote culture (5 × 10^7 ^cells/ml) in the log phase. Parasites were harvested by centrifugation (5,000 × *g *for 20 min at 4°C) and washed four times in PBS. Cells were resuspended in 1.0 ml of Milli-Q water in the presence of 10 μM of the protease inhibitors *trans*-epoxysuccinyl-L-leucylamido-(4-guanidino)butane (E-64; Sigma-Aldrich) and tosyl-lysylchloromethane (TLCK; Sigma-Aldrich) and disrupted by three cycles of freezing at -20°C and thawing. The insoluble material was removed by centrifugation (20,000 × *g *for 20 min at 4°C) and the supernatant, referred to hereafter as enzyme extract, was immediately used for the assays or stored at -80°C. Protein content was determined by the Bradford method.

### Assay of peptidase activity

*T. cruzi *aminopeptidase activity was assayed on the fluorogenic substrates *L*-Leu-7-amido-4-methylcoumarin (Leu-AMC), *N*-carbobenzoxy-Leu-AMC (N-Cbz-Leu-AMC), *L*-Pro-AMC (Pro-AMC) and Asp-AMC, which were purchased from Sigma-Aldrich. Enzyme activity was determined by measuring the fluorescence of AMC released by hydrolysis of the substrates as described previously [28]. Assays were performed by incubating 1.0 μl of enzyme extract (2.5 μg of protein) or 30 ng of purified LAPTc, as specified, for 15 min at the desired temperature in 100 μl final volume of reaction buffer (25 mM Tris-HCl, pH 7.5) in the presence of 20 μM fluorogenic substrate. Enzymatic activity is expressed in mU/mg, where 1 U represents 1 mmol of released AMC/min. In-gel leucyl aminopeptidase activity of either enzyme extract (5 μg) or purified LAPTc (0.1 μg) was performed on 8% SDS-PAGE essentially as described previously [29]. Samples were solubilized in Laemmli buffer containing 0.1 or 0.01% SDS and subjected to electrophoresis at 4°C under non-reducing conditions without prior heating to 100°C. Next, the gel was washed 4 times in reaction buffer, 20 min each time, and incubated at 37°C for up to 30 min in the presence of 50 μM Leu-AMC. To determine kinetic parameters, purified LAPTc was incubated in reaction buffer with variable Leu-AMC concentrations (1 to 100 μM) and the enzyme reaction was carried out as described above. Kinetic parameters were determined by fitting the rate data to the Michaelis-Menten equation. *k*_cat _was calculated by the equation *k*_cat _= *V*_max_/[E]_0_, where [E]_0 _represents the active enzyme concentration.

### LAPTc purification and electrophoretic analysis

*T. cruzi *peptidase with specificity for Leu-AMC was purified from freshly prepared enzyme extract by fast liquid chromatography. Enzyme extract (1 ml; 5.6 mg of protein) was buffered with 25 mM Tris-HCl pH 7.5, filtered through a 0.22 μm membrane and applied to a DEAE-Sepharose CL-6B (Sigma-Aldrich) column (5 cm × 1 cm), previously equilibrated with 25 mM Tris-HCl, pH 7.5. After washing the column, bound proteins were eluted with a linear gradient performed in the same buffer from 0.3 to 0.65 M NaCl for 30 min, and then from 0.66 to 1.0 M NaCl for 10 min at a 0.5 ml/min flow rate. Fractions of 0.25 ml were collected on ice, and an aliquot of each fraction was assayed with Leu-AMC. Enzymatically active fractions were pooled and concentrated to 250 μl with a Centricon 100 (Amicon) at 4°C. The solution was then submitted to size exclusion chromatography on a Superose 6 HR 10/30 column (GE Healthcare) isocratically perfused with 25 mM Tris-HCl, 150 mM NaCl, pH 7.5, at a 0.3 ml/min flow rate for 80 min, and calibrated with bovine serum albumin (67 kDa), aldolase (158 kDa), catalase (232 kDa), ferritin (440 kDa), and thyroglobulin (669 kDa). Each 250- μl fraction was instantly stored on ice until enzyme activity assay, and the active ones were pooled and concentrated to 100 μl as above. Then, 30 ng of the purified protein were subjected to 8% SDS/PAGE under non-reducing conditions without previous boiling, and the gel silver-stained. The presence of interchain disulfide bonds, the molecular mass and the oligomeric structure of the enzyme were evaluated by electrophoresis as described previously [36].

### Identification of *T. cruzi *aminopeptidase by peptide mass fingerprinting

The purified native protein (2 μg) was digested with trypsin (Promega, Madson, Wis) at 37°C for 12 h for peptide mass fingerprinting as described [45]. The digested sample was applied to a MALDI-TOF Reflex mass spectrometer (Bruker Daltonics). Experimentally measured peptide molecular masses were subjected to a protein identity search against the nonredundant database of the National Center for Biotechnology Information (NCBI) via BioTool 2.0 (Bruker Daltonics) and the Mascot program http://www.matrixscience.com. The following parameters were used for database searches: monoisotopic mass accuracy up to 0.2 Da for internally calibrated spectra; up to one missed cleavage site; carbamidomethylation of cysteine as fixed chemical modification; and oxidation of methionine as variable chemical modification. The protein was identified as a leucyl aminopeptidase (LAP; accession number EAN97960).

### Phylogenetic relationship of LAPTc with other LAPs

Twenty-nine sequences were selected from the nonreduntant (NR) protein database of NCBI after a search for M17 family members from different organisms under the following accession numbers: EAN97960.1, EAN99056.1 and EAN87580.1 (*T. cruzi *1, 2 and 3, respectively), EAN79621.1 and AAX70152.1 (*Trypanosoma brucei *1 and 2, respectively), CAJ02694.1, CAJ06706.1 and AAL16097.1 (*Leishmania major *1, 2 and 3, respectively), CAM36610.1 (*Leishmania braziliensis*), AAL16095.1 (*Leishmania amazonensis*), AAL16096.1 (*Leishmania donovani*), AAD17527.1 (*Homo sapiens*), XP_001162589.1 (*Pan troglodytes*), NP_077754.2 (*Mus musculus*), NP_001011910.1 (*Rattus norvegicus*), NP_252520.1 (*Pseudomonas aeruginosa*), ZP_01789367.1 (*Haemophilus influenzae*), YP_672349.1 (*Escherichia coli*), YP_001217999.1 (*Vibrio cholerae*), CAC31245.1 (*Mycobacterium leprae*), NP_216729.1 (*Mycobacterium tuberculosis*), NP_194821.1 (*Arabidopsis thaliana*), CAN66364.1 (*Vitis vinifera*), NP_001066684.1 (*Oryza sativa*), CAA69614.1 (*Solanum lycopersicum*), XP_744254.1 (*Plasmodium chabaudi*), EAA21300.1 (*Plasmodium yoelii*), XP_001348613.1 (*Plasmodium falciparum*) and XP_001615930.1 (*Plasmodium vivax*). Sequence alignments were conducted with the ClustalX software package [46]. Phylogenetic analysis and statistical neighbor-joining bootstrap tests of the phylogenies were performed with the Mega package [47].

### Cloning and expression of the recombinant enzyme (rLAPTc)

According to the sequence of the gene encoding LAPTc (gene ID Tc00.1047053508799.240; http://www.tcruzidb.org/tcruzidb/home-ori.jsp), specific primers (forward primer 5' -CTAGTGA**CATATG**AACAGACCTCCTGCTACA - 3', NdeI site in bold, and reverse primer 5' - TAGTGA**CTCGAG**TTATCGTAAATTACGAAGATATTCC - 3', XhoI in bold) were designed and used to amplify the *laptc *open reading frame from *T. cruzi *genomic DNA. The PCR product was cloned into the pCR2.1-TOPO vector. The clone was digested with NdeI and XhoI and the 1563 bp full-length fragment was cloned into the pET-19b expression vector (Novagen). Gene cloning was confirmed by DNA sequencing. The N-terminal His-tagged rLAPTc was produced in *E. coli *BL21(DE3) through 1.0 mM IPTG induction at 20°C over 5 h. Cells were harvested by centrifugation, resuspended in lysis buffer (20 mM Tris-HCl, 500 mM NaCl, 5 mM imidazole, 0.2% lysozyme, pH 7.9), submitted to sonication on ice and centrifuged at 15,000 × *g *for 10 min at 4°C. Then, the supernatant was submitted to affinity chromatography on a nickel column and rLAPTc was eluted with 400 mM imidazole and further purified by size exclusion chromatography on a Superose 6 HR 10/30 column as described above. rLAPTc, the main peak of activity obtained after the last purification step, was used for enzymatic assays and analyzed by 8% PAGE in the presence of 0.1 or 0.01% SDS, followed by Coomassie staining of the gel.

### Molecular organization assay: analytical ultracentrifugation and light scattering

Sedimentation velocity experiments were performed using a Beckman XL-I analytical ultracentrifuge and an AN-60 TI rotor (Beckman Coulter). Experiments were carried out at 10°C for rLAPTc, obtained after affinity chromatography, at 170, 56 and 10 μM in 25 mM Tris pH 8.0, 150 mM NaCl, corresponding to absorbancies at 280 nm of 3.5, 1.2 and 0.2, respectively. A volume of 110 μl (for the most concentrated sample) or 420 μl was loaded into 0.3 or 1.2 cm path cells and centrifuged at 42,000 rpm. Scans were recorded every 6 min, overnight, at 295 and 285 nm and by interference. We used the Sednterp software (free available at http://www.jphilo.mailway.com) to estimate the partial specific volume of the polypeptide chain, $\bar{v}$, the solvent density, *ρ *= 1.00667 g/ml, and the solvent viscosity, *η *= 1.335 mPa.s, at 10°C. Sedimentation profiles were analyzed by the size-distribution analysis of Sedfit (freely available at http://www.analyticalultracentrifugation.com). In Sedfit, finite element solutions of the Lamm equation for a large number of discrete, independent species, for which a relationship between mass, sedimentation and diffusion coefficients, *s *and *D*, is assumed, are combined with a maximum entropy regularization to represent a continuous size-distribution [48]. We used typically 200 generated data sets, calculated on a grid of 300 radial points and using fitted frictional ratio for sedimentation coefficients comprised between 1 and 50 S. For the regularization procedure a confidence level of 0.68 was used.

The molecular mass of LAPTc in solution was also determined by size exclusion chromatography coupled to multiangle laser light scattering (SEC-MALLS) and refractometry (RI). rLAPTc (20 μl), purified by affinity chromatography as above, at 170 μM (10 mg/ml) in 25 mM Tris-HCl, pH 7.5, 100 mM NaCl, was injected in a KW 804 column preceded by a guard column (Shodex), equilibrated in the same solvent, at 20°C with a flow rate of 0.5 ml/min. Protein concentration was measured on-line by refractive index (RI) measurements using an Optilab rEX (Wyatt Technology) and considering ∂n/∂c = 0.186 ml/g. On-line MALLS detection was performed with a miniDAWN TREOS detector (Wyatt Technology) using laser emitting at 658 nm. Data were analyzed and weight-averaged molar masses were calculated using the ASTRA software (Wyatt Technology Corp.). Elution profiles (left ordinate axis) were monitored by RI. The molecular mass distribution (right ordinate axis) was determined from combined MALLS and RI data.

### Assay of optimal pH and temperature for activity and thermostability of LAPTc

The optimal pH for activity of both endogenous and recombinant LAPTc was determined as described above in 50 mM acetic acid-50 mM MES-50 mM Tris-HCl buffer adjusted to the desired pH. To assay the optimal temperature for aminopeptidase activity, reactions took place at 20, 25, 30, 37, 40, 50, 60, 70, 80 or 100°C in reaction buffer. Enzyme thermostability was assayed by incubating the purified proteins at the same temperatures for either 15 or 240 min in reaction buffer before the aminopeptidase activity assay on Leu-AMC. An 8% SDS-PAGE analysis of the molecular organization of the native or recombinant LAPTc followed. PAGE was performed in the presence of 0.1 or 0.01% SDS without previous boiling of either protein.

### Inhibition pattern and cation dependence of LAPTc

Different concentrations of tosyl-lysylchloromethane (TLCK), bestatin, EDTA, L-*trans*-epoxysuccinylleucylamido-(4-guanidino) butane (E-64), phenylmethylsulfonyl fluoride (PMSF), 1,10-phenanthroline, leupeptin, or phosphoramidon were incubated with 50 ng of purified LAPTc in 100 μl reaction buffer for 20 min at room temperature before the substrate was added. Enzymatic reactions were monitored as described above. All inhibitors were from Sigma-Aldrich. To assess the effects of cations on enzymatic activity, purified LAPTc was incubated in reaction buffer containing 10 mM EDTA or 250 μM 1,10-phenanthroline for 30 min at room temperature. After extensive dialysis against reaction buffer at 4°C, 20 μM Leu-AMC (final concentration) and AlCl_3_, CaCl_2_, FeCl_2_, CoCl_2_, MgCl_2_, MnCl_2_, or ZnCl_2 _(final concentration, 0.4 mM) were added to the reaction system, followed by a 15 min incubation at 37°C. Hydrolysis of the substrate was measured as described above. Controls consisted of enzymatic reactions carried out either without EDTA or 1,10-phenanthroline treatments or in the absence of cations.

### Analysis of expression and immunocytolocalization of LAPTc

One 4-month-old female rabbit was immunized with 13 μg of purified LAPTc emulsified in complete Freund's adjuvant followed by two biweekly boosters with the enzyme in incomplete Freund's adjuvant. Four days after the last booster, serum was collected and Western blotting monitored the presence of anti-LAPTc specific antibodies. To assay the expression of LAPTc by *T. cruzi *epimastigotes, total parasite proteins were subjected to 8% SDS-PAGE with or without previous heating to 100°C and transferred to a nitrocellulose membrane. The membrane was blocked by incubation in 5% (w/v) non-fat milk/PBS for 3 h at room temperature. Blots were incubated in 1% non-fat milk/PBS for 2 h in the presence of either pre-immune or immune serum diluted to 1:400, followed by extensive washing in PBS. Then, the membranes were incubated with alkaline phosphatase-conjugated anti-rabbit IgG diluted to 1:2000, washed in PBS and the immunocomplexes revealed with 5-bromo-4-chloro-3-indolyl-1-phosphate/Nitro Blue Tetrazolium (Promega). For immunofluorescence, epimastigotes, amastigotes and trypomastigotes of *T. cruzi *were fixed overnight at 4°C with 3.7% formaldehyde, air-dried on poly-L-lysine-coated glass slides, permeabilized with 0.2% (v/v) Triton X-100 and incubated with pre-immune or anti-LAPTc serum (1:50 in 1% non-fat milk/PBS) for 2 h at room temperature. After extensive washing in 1% non-fat milk/PBS, cells were incubated with Alexa 488-conjugated goat anti-rabbit IgG for 1 h. This was followed by washing and staining parasite DNAs with 5 μg/ml 4,6-diamino-2-phenylindole (DAPI) for 5 min. Glass slides were washed, mounted and observed with a Leica TCS SP5 confocal microscope (Leica Microsystems, Wetzlar, Germany).

## Authors' contributions

GCR and TSG were involved in all of the experimental and theoretical work. EF, MR and CE performed the analytical centrifugation and light scattering experiments and commented on the manuscript. HA and TCA helped with data analysis and sequence alignment. KCA, MML and RSN participated in the biochemical studies. BMR and CRF were involved in the cloning and kinetic experiments. IMDB participated in the design and coordination of the experiments. JMS conceived of the study, participated in its design and coordination and drafted the manuscript. All authors have read and approved the final manuscript.
